# Mammalian monocarboxylate transporter 7 (MCT7/Slc16a6) is a novel facilitative taurine transporter

**DOI:** 10.1016/j.jbc.2022.101800

**Published:** 2022-03-05

**Authors:** Kei Higuchi, Koki Sugiyama, Ryuto Tomabechi, Hisanao Kishimoto, Katsuhisa Inoue

**Affiliations:** Department of Biopharmaceutics, School of Pharmacy, Tokyo University of Pharmacy and Life Sciences, Hachioji, Tokyo, Japan

**Keywords:** monocarboxylate transporter 7, taurine, facilitative transport, efflux, membrane transport, amino acid transport, intestinal epithelium, transporter, protein–protein interaction, AAVS, adeno-associated virus integration site, BiFC, bimolecular fluorescence complementation, EGFP, enhanced green fluorescent protein, MCT7, monocarboxylate transporter 7, MCTs, monocarboxylate transporters, PAT1, H^+^-coupled amino acid transporter 1, SLC16A, solute carrier family 16A, TAUT, Na^+^-coupled taurine transporter, VC, *C*-terminal Venus-fragment, VN, *N*-terminal Venus-fragment, VRAC, volume-regulated anion channel

## Abstract

Monocarboxylate transporter 7 (MCT7) is an orphan transporter expressed in the liver, brain, and in several types of cancer cells. It has also been reported to be a survival factor in melanoma and breast cancers. However, this survival mechanism is not yet fully understood due to MCT7’s unidentified substrate(s). Therefore, here we sought to identify MCT7 substrate(s) and characterize the transport mechanisms by analyzing amino acid transport in HEK293T cells and polarized Caco-2 cells. Analysis of amino acids revealed significant rapid reduction in taurine from cells transfected with enhanced green fluorescent protein-tagged MCT7. We found that taurine uptake and efflux by MCT7 was pH-independent and that the uptake was not saturated in the presence of taurine excess of 200 mM. Furthermore, we found that monocarboxylates and acidic amino acids inhibited MCT7-mediated taurine uptake. These results imply that MCT7 may be a low-affinity facilitative taurine transporter. We also found that MCT7 was localized at the basolateral membrane in polarized Caco-2 cells and that the induction of MCT7 expression in polarized Caco-2 cells enhanced taurine permeation. Finally, we demonstrated that interactions of MCT7 with ancillary proteins basigin/CD147 and embigin/GP70 enhanced MCT7-mediated taurine transport. In summary, these findings reveal that taurine is a novel substrate of MCT7 and that MCT7-mediated taurine transport might contribute to the efflux of taurine from cells.

Monocarboxylate transporters (MCTs) belong to the solute carrier family 16A (SLC16A) that comprises 14 members. MCTs (MCT1–4) catalyze H^+^-coupled transport of metabolically important monocarboxylates such as lactate and contribute to maintenance of intracellular pH (1). Typical MCTs are known to mediate the bidirectional transport of substrates and protons depending on their concentration gradients across the plasma membrane (2, 3). MCT1 and MCT4 in cancer cells are related to tumor growth and proliferation by mediating the influx and efflux of lactate and protons (4). Functional expression of MCTs is supported by several ancillary proteins such as basigin/CD147 and embigin/GP70 (5). These ancillary proteins interact with MCTs at the plasma membrane and regulate membrane localization and function. Apart from prototypical MCTs, other members transport not only monocarboxylates but also zwitterionic metabolites as substrates. For instance, MCT12/SLC16A12 transports intracellular creatine across the plasma membrane in a H^+^-independent manner (6, 7). MCT9/SLC16A9 also functions as a H^+^-independent carnitine efflux transporter (8). However, the substrates of several MCTs have not been identified, and the physiological role has not been fully understood.

Mammalian monocarboxylate transporter 7 (MCT7/SLC16A6) is an orphan transporter of the SLC16A family whose substrates and transport mechanisms are yet to be characterized. An ortholog of mammalian MCT7 in zebrafish (encoded by *Slc16a6a*) is reported to transport a ketone body, β-hydroxybutyrate, and the loss of gene function was shown to cause steatosis of the liver (9). Exogenous gene expression of human *SLC16A6* in the liver, instead of *Slc16a6a*, enabled liver recovery from the steatosis. Based on this report, MCT7 is classified as a transporter for ketone bodies; however, the physiological substrates of mammalian MCT7 remain unclarified. In humans, MCT7 is primarily expressed in the liver, brain, and endocrine pancreas (10). In addition, intestine and colon are the expression sites of MCT7 in rodents (11). Interestingly, MCT7 is highly expressed in melanoma cells, and single nucleotide polymorphisms are related to cutaneous melanoma survival (12). Considering that ketogenesis occurs primarily in the mitochondria of hepatocytes, such wide expression of MCT7 implies multiple physiological roles in the body. Therefore, identification of mammalian MCT7 substrate(s) is crucial in understanding functionality.

The purpose of this study was to identify MCT7 substrates and characterize transport mechanisms. Substrate identification focused on efflux profiles of endogenous amino acids from the MCT7-transfected cells. Taurine emerged as a novel substrate of MCT7. We then examined MCT7-dependent taurine transport characteristics and localization using HEK293T cells and polarized Caco-2 cells. Furthermore, MCT7 regulation by ancillary proteins CD147 and GP70 was also investigated.

## Results

### Decrease of taurine content in MCT7-transfected cells

**Figure 1:**
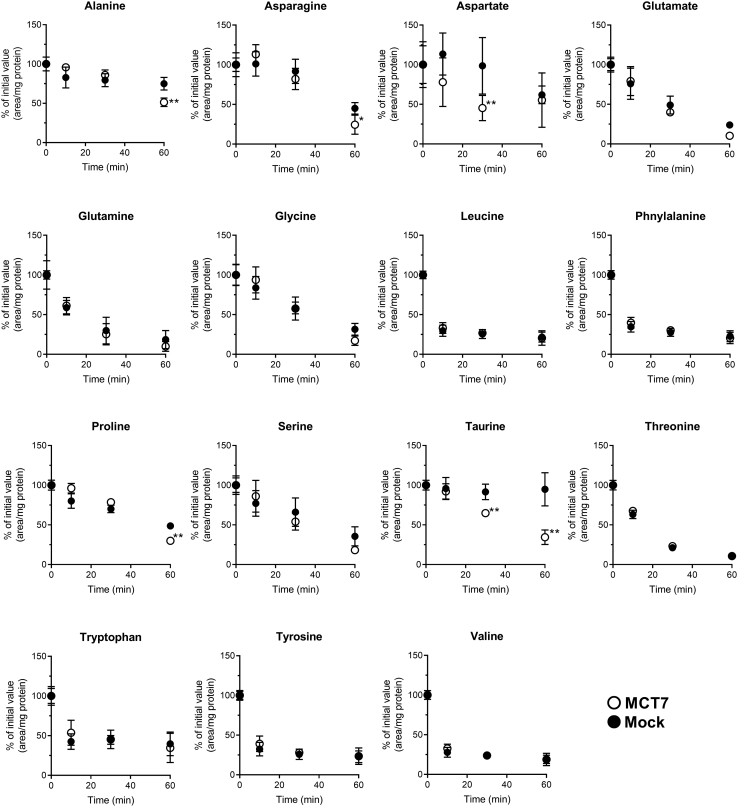
**MCT7 causes a rapid decrease of intracellular taurine level.** HEK293T cells were transfected with EGFP-tagged MCT7 (○) or empty-vector (mock) (•). The transfected cells were incubated in Na^+^-free buffer (pH 7.4) at 37 °C for designed time. After the incubation, the amino acids contents were measured by LC-MS/MS and the obtained chromogram area is correlated by protein amount. The values were expressed as % of initial value. Each point represents the mean ± S.D. (n = 7). ∗∗ *p* < 0.01 and ∗ *p* < 0.05, compared with the corresponding mock-transfected cells by two-way ANOVA with Sidak's multiple comparisons test. EGFP, enhanced green fluorescent protein; MCT7, monocarboxylate transporter 7.

Since some MCTs recognize zwitterionic metabolites and amino acid derivatives (7, 8, 13), we first examined efflux profiles of amino acids in HEK293T cells expressing enhanced green fluorescent protein (EGFP)-tagged MCT7. Efflux of endogenous amino acids was measured using Na^+^-free buffer to suppress the Na^+^-dependent reuptake activity. After more than 30 min of incubation, levels of alanine, asparagine, aspartic acid, proline, and taurine in MCT7-transfected cells were lower than mock-transfected cells (Fig. 1). In comparison to the other amino acids, only taurine content showed a significant decrease in MCT7-transfected cells, whereas not in mock-transfected cells. This efflux profile suggests that MCT7 is involved in the regulation of intracellular taurine levels.

### Characteristics of taurine transport by MCT7

**Figure 2:**
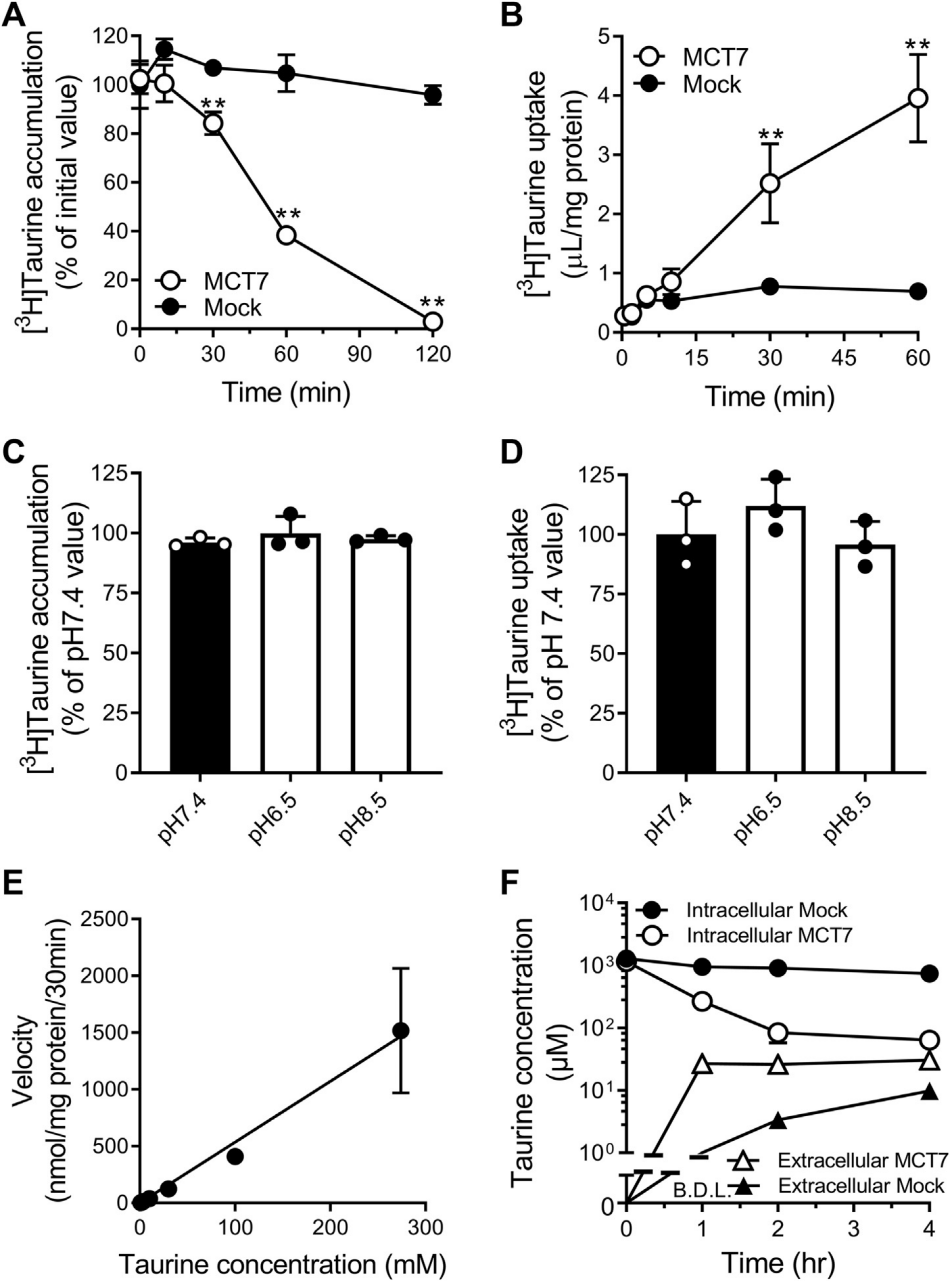
**MCT7 mediates a bidirectional taurine transport.***A* and *C*, efflux of [^3^H]taurine by HEK293T cells expressing EGFP-tagged MCT7 (MCT7) or empty-vector (mock). [^3^H]taurine was loaded to the cells by incubation in NaCl buffer for 30 min. The cells were incubated in Na^+^-free buffer (pH 7.4) for designed time or in the pH-modified buffer (pH 6.5 or pH 8.5) for 30 min. The residual [^3^H]taurine in the cells was measured. *B* and *D*, [^3^H]taurine uptake (5 μM) was measured in Na^+^-free buffer (pH 7.4) for designed time or in the pH modified buffer (pH 6.5 or pH 8.5) for 30 min. *E*, concentration dependence of [^3^H]taurine uptake. The uptake was measured at designed concentration (1–270 mM). Velocity of MCT7-mediated uptake was calculated by subtracting the taurine uptake in mock-transfected cells from EGFP-tagged MCT7 transfected cells. *F*, intracellular and extracellular concentration of taurine in MCT7- or mock-transfected cells. The cells were incubated in Na^+^-free buffer (pH 7.4) for designed time. The residual taurine in the cells and effluxed taurine in the buffer were measured by LC-MS/MS. Each point represents the mean ± S.D. (n = 3). ∗∗ *p* < 0.01, compared with the corresponding mock-transfected cells by two-way ANOVA with Sidak's multiple comparisons test or one-way ANOVA with Tukey’s multiple comparison test. EGFP, enhanced green fluorescent protein; MCT7, monocarboxylate transporter 7.

To clarify whether taurine is directly transported by MCT7, we performed uptake and efflux experiments using HEK293T cells expressing EGFP-tagged MCT7. For efflux experiments, [^3^H]taurine was loaded in the cells using a buffer containing Na^+^, and then, the cells were incubated in Na^+^-free buffer. The loaded [^3^H]taurine amount of MCT7-transfected cells (398 ± 18 μl/30 min/mg protein) was similar to levels in mock-transfected cells (371 ± 13 μl/30 min/mg protein). After incubation in Na^+^-free buffer for 120 min, [^3^H]taurine amount in the MCT7-transfected cells was reduced by 95% of the initial amount, whereas no changes were observed in the mock-transfected cells (Fig. 2*A*). Next, we examined whether MCT7 mediates taurine uptake into cells. In Na^+^-free buffer, [^3^H]taurine was taken up into the MCT7-transfected cells with time (Fig. 2*B*). The uptake in MCT7-transfected cells increased in a linear fashion for a duration of 30 min, whereas uptake in mock-transfected cells plateaued at 5 min. We also examined the ability of untagged MCT7 to transport taurine *via* uptake and efflux studies (Fig. S1). However, the transport activities of untagged MCT7 were lower than those of EGFP-tagged MCT7 (Fig. 2, *A* and *B*). Therefore, we used the EGFP-tagged transporter for evaluating the transport function of MCT7. Furthermore, the uptake and efflux of [^3^H]taurine by MCT7 did not change under acidic (pH 6.5) and alkaline pH (pH 8.5) buffer conditions (Fig. 2, *C* and *D*). The concentration dependence of the taurine uptake was examined, and we observed no saturation in concentrations between 1 and 270 mM (Fig. 2*E*). These data suggest that MCT7 can directly transport taurine as an ultra-low affinity substrate in a Na^+^- and pH-independent manner and mediate the influx and efflux of taurine. We also determined the intracellular and extracellular concentrations of taurine in MCT7- and mock-transfected cells at equilibrium (Fig. 2*F*). The intracellular taurine concentration was calculated, assuming that the intracellular volume is 6.5 μl/mg protein (14). After 4 h of incubation, at which the taurine concentration seemed to reach equilibrium, the intracellular-to-extracellular taurine concentration ratio in MCT7-transfected cells was 2.1, whereas that in mock-transfected cells was 76. The calculated value in MCT7-transfected cells indicated that the taurine concentration in the cytosol was comparable with the extracellular concentration at equilibrium, considering that a part of intracellular taurine is distributed in subcellular compartments such as mitochondria (15). These data suggest that MCT7 mediates the facilitated diffusion of taurine.

### Functional regulation of MCT7 by ancillary proteins CD147 and GP70

**Figure 3:**
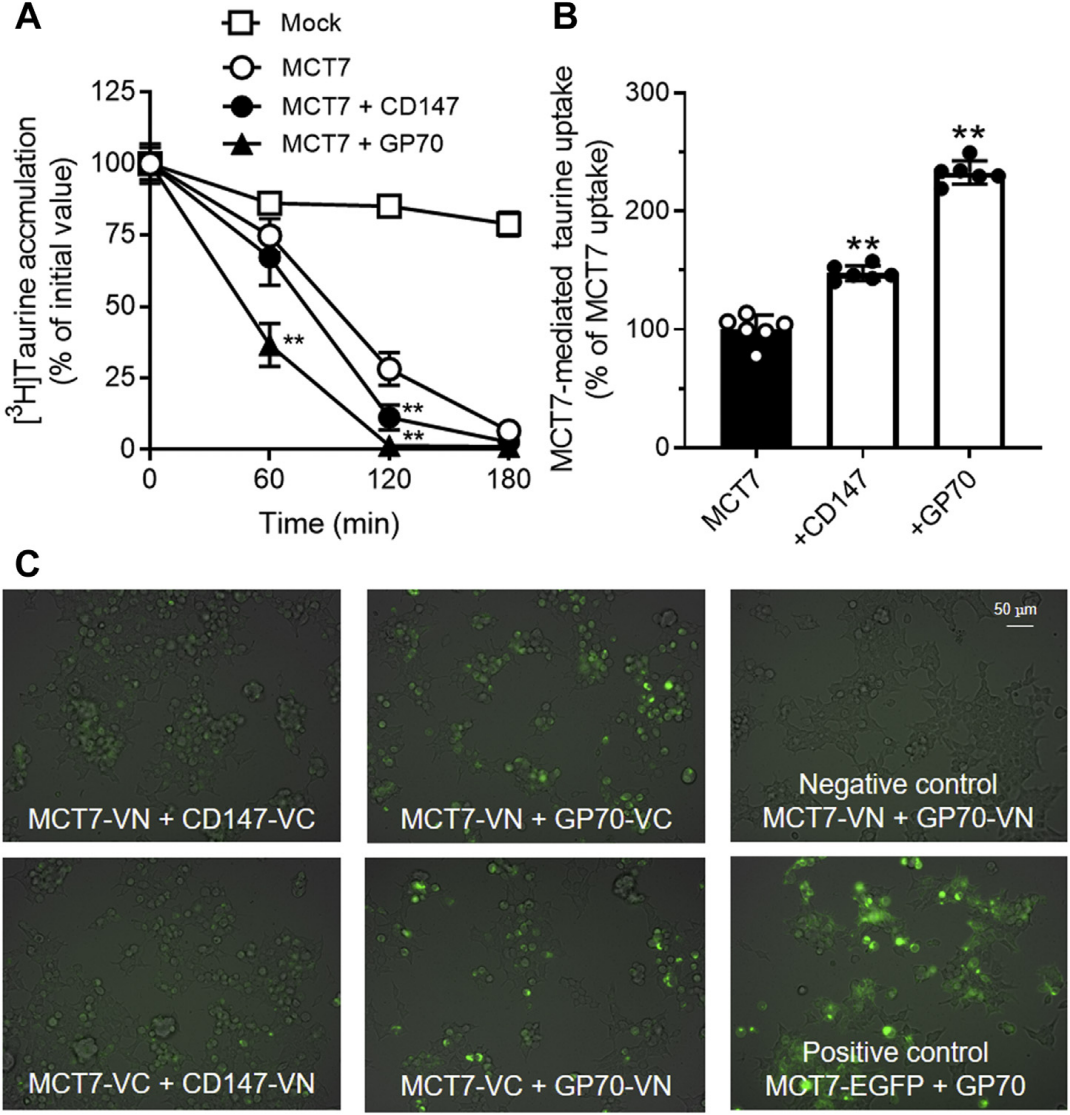
**Ancillary proteins CD147 and GP70 interact with MCT7 and regulate the function.** HEK293T cells were transfected with expressing EGFP-tagged MCT7 and CD147 or GP70. *A*, MCT7-mediated [^3^H]taurine efflux was measured. [^3^H]taurine was loaded into those cells by incubation with NaCl buffer. Those cells were incubated in Na^+^-free buffer (pH 7.4) for designed time. The residual [^3^H]taurine in the cells was measured. Each point represents the mean ± S.D. (n = 3). *B*, [^3^H]taurine uptake (5 μM) was measured in Na^+^-free buffer (pH 7.4) for 2 min. MCT7-mediated uptake was calculated by subtracting the uptake amount of mock-transfected cells from that of MCT7-transfected cells. Each bar represents the mean ± S.D. (n = 6). ∗∗ *p* < 0.01, compared with the corresponding only MCT7-transfected cells by one-way ANOVA with Tukey’s multiple comparison test. *C*, Bi-FC assay of interaction between MCT7 and ancillary proteins. VN and VC are the *N*- and *C*-terminal fragments of Venus fluorescent protein, respectively. HEK293T cells were transfected with MCT7-VN or MCT7-VC and ancillary proteins-VN or -VC. EGFP-tagged MCT7 was used as a positive control. After 48-hr transfection, Venus fluorescent signals were detected by a fluorescence microscopy. The obtained fluorescent images were merged with phase contrast images. EGFP, enhanced green fluorescent protein; MCT7, monocarboxylate transporter 7; VC, C-terminal Venus-fragment; VN, N-terminal Venus-fragment.

To examine whether ancillary proteins CD147 and GP70 influence MCT7 functionality, we performed efflux and uptake studies using HEK293T cells expressing EGFP-tagged MCT7 and the ancillary proteins. Loaded [^3^H]taurine rapidly decreased in the cells co-expressing MCT7 and each ancillary protein compared with the MCT7-transfected cells (Fig. 3*A*). In parallel with the result, [^3^H]taurine uptake *via* MCT7 was increased by 148% and 233% in tandem with expression of CD147 and GP70, respectively (Fig. 3*B*). Interestingly, CD147 and GP70 effect on taurine uptake was not observed in the mock-transfected cells: Mock (0.18 μl/2 min/mg protein), CD147-transfected cells (0.20 μl/2 min/mg protein), and GP70-transfected cells (0.17 μl/2 min/mg protein). Next, we examined the interaction of MCT7 with ancillary proteins by bimolecular fluorescence complementation (BiFC) assay (16). Venus fluorescence was detected in the cells co-expressing *N*-terminal Venus-fragment–tagged MCT7 (MCT7-VN) and *C*-terminal Venus-fragment–tagged ancillary proteins (CD147-VC and GP70-VC), but not in the cells co-expressing MCT7-VN and *N*-terminal Venus-fragment–tagged ancillary protein (Fig. 3*C*). The fluorescence was also observed when using the cells co-expressing *C*-terminal Venus-fragment–tagged MCT7 (MCT7-VC) and *N*-terminal Venus-fragment–tagged ancillary proteins (CD147-VN and GP70-VN). These data suggest MCT7 can be regulated by the interaction with ancillary proteins CD147 and GP70.

### Enhancement of taurine permeation by MCT7 across Caco-2 cells

**Figure 4:**
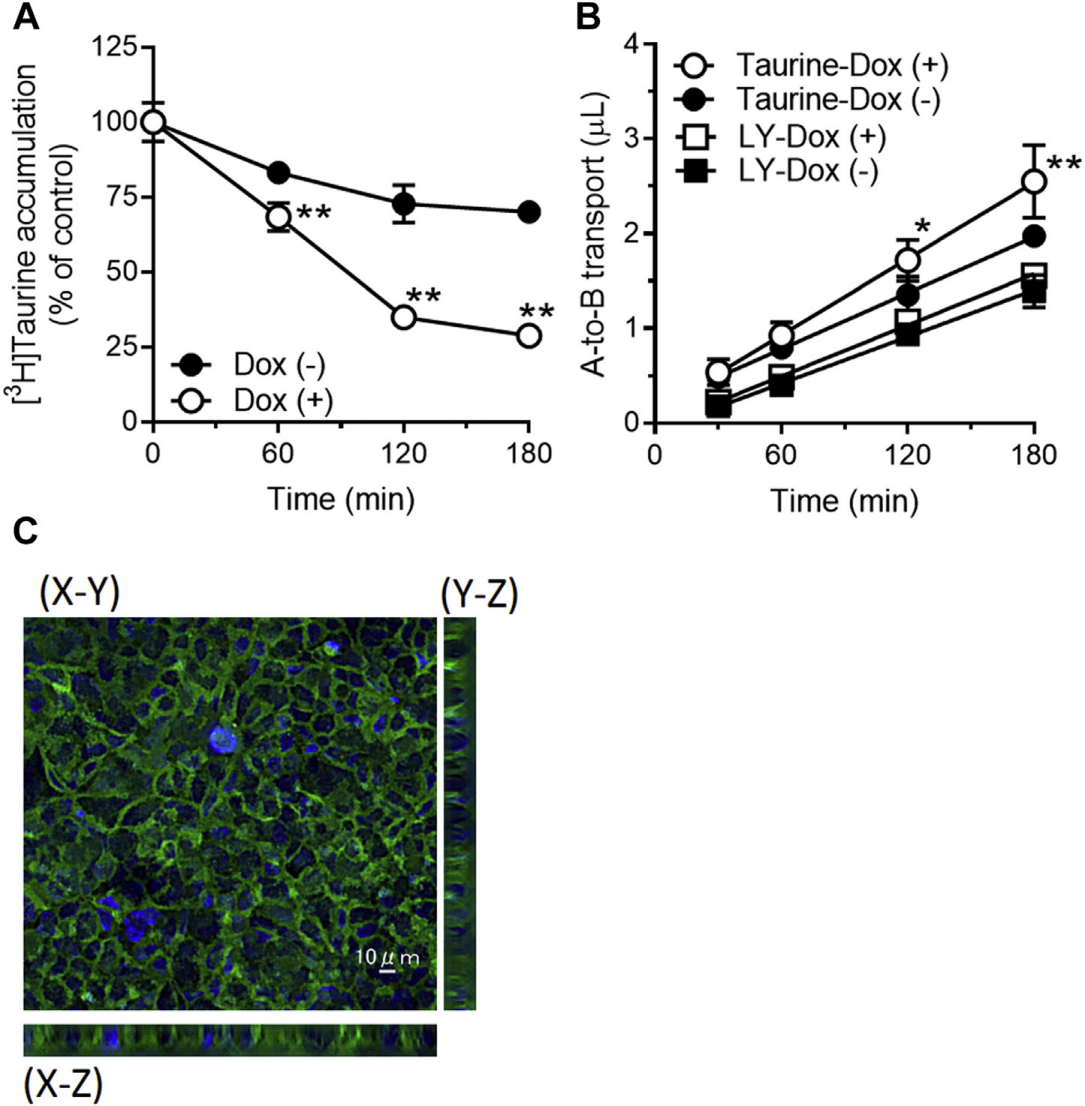
**MCT7 enhances taurine penetration across Caco-2 cells.***A*, efflux of [^3^H]taurine by Caco-2-Tet-MCT7 cells inducing MCT7. Caco-2-Tet-MCT7 cells were cultured on 48-well plate and then treated with doxycycline at 5 μg/ml and sodium butyrate at 10 mM (Dox [+]) or sodium butyrate at 10 mM (Dox [-]) for 48 h to induce MCT7 expression. [^3^H]taurine was loaded into those cells by incubation with NaCl buffer. Those cells were incubated in Na^+^-free buffer (pH 7.4) for designed time. The residual [^3^H]taurine in the cells was measured. *B*, apical to basal (A-to-B) transport of [^3^H]taurine and lucifer yellow (LY). Caco-2-Tet-MCT7 cells were cultured on a Transwell membrane. After 21 days of culture, the cells were treated with doxycycline as description above. Transport of [^3^H]taurine (5 μM) and LY (500 μM) from apical to basal chamber were measured. *C*, localization of induced MCT7 in Caco-2-Tet-MCT7 cells cultured on a Falcon cell culture insert membrane. The nuclei were stained by Hoechst33342. MCT7 (*green*) and nuclei (*blue*) were observed by a confocal fluorescence microscopy. Each point represents the mean ± SD (n = 3). ∗∗ *p* < 0.01 and ∗ *p* < 0.05, compared to the corresponding Caco-2-Tet-MCT7 cells treated with only sodium butyrate by two-way ANOVA with Sidak's multiple comparisons test. MCT7, monocarboxylate transporter 7.

To examine whether MCT7 plays a role in taurine intestinal absorption, we prepared Caco-2 cells in which EGFP-tagged MCT7 expression was driven by the conditional Tet-on promoter (Caco-2-Tet-MCT7). We first attempted to detect the [^3^H]taurine efflux activity of MCT7 in Caco-2-Tet-MCT7 cells treated with only doxycycline, but we observed no activity (Fig. S2). Next, we treated Caco-2-Tet-MCT7 cells with sodium butyrate, which is known to enhance doxycycline inducibility (17, 18), in parallel with doxycycline treatment. As a result, MCT7 expression levels in Caco-2-Tet-MCT7 cells were increased following the treatment (Fig. S2), and [^3^H]taurine efflux from the MCT7-induced cells was greater than that from the noninduced cells (Fig. 4*A*). Next, we examined the effect of MCT7 expression on the taurine penetration across the polarized Caco-2-Tet-MCT7 cells. The apical-to-basal transport of taurine was enhanced by induction of MCT7 expression, whereas that of lucifer yellow, a marker of paracellular permeability, remained unchanged (Fig. 4*B*). Further the induced MCT7 was mainly localized at basolateral membrane of Caco-2-Tet-MCT7 cells (Fig. 4*C*). These data suggest that MCT7 may function as a taurine releaser at basolateral membrane of polarized intestinal epithelial cells and contribute to taurine intestinal absorption.

### Inhibitory effect of substrates/inhibitors for TAUT, PAT1, and MCTs on MCT7-mediated taurine uptake

Na^+^-coupled taurine transporter (TAUT)/SLC6A6 and H^+^-coupled amino acid transporter 1 (PAT1)/SLC36A1 are known to function as Na^+^- and H^+^-coupled taurine transporters, respectively, in the intestine (19). Therefore, we examined the effect of substrates/inhibitors for these transporters on MCT7-mediated taurine uptake (Table 1). We also checked the effect of other MCTs’ substrates and several amino acids on the uptake. For TAUT inhibitors, only homocysteic acid inhibited MCT7-mediated taurine uptake of 26%, but L-alanine and L-proline, which are substrates of PAT1, did not inhibit the uptake. For amino acids, L-glutamate and L-aspartate decreased the uptake to 33% and 67%, respectively. Monocarboxylates had an inhibitory effect on the uptake. Interestingly, the inhibitory effect of lactate, a prototypical MCTs’ substrate, was weaker than that of butyrate or β-hydroxybutyrate; lactate caused only 25% inhibition. In substrates of other MCTs, L-carnitine also had a weak inhibitory effect on the uptake. We also examined the inhibitory effect of two ketone bodies (acetone and acetoacetate) on MCT7-mediated taurine uptake. Acetoacetate showed a weak inhibitory effect, whereas acetone showed no inhibitory effect. Ketone bodies were unlikely to be a specific substrate of MCT7.

**Table 1:** Effect of MCTs, TAUT substrates/inhibitors on [^3^H]taurine uptake by MCT7

Classification		MCT7-mediated uptake	
		Substrates/inhibitors	(% of control)
		Control	100 ± 5
TAUT		β-alanine	102 ± 14
		GABA	108 ± 24
		homocysteic acid	25.6 ± 13.7^a^
PAT1		L-alanine	102 ± 17
		L-proline	108 ± 26
Amino acids		L-asparagine	99.3 ± 7.5
		L-asparatate	66.7 ± 8.7^b^
		L-glutamate	32.7 ± 3.3^a^
		L-phenylalanine	102 ± 9.4
MCTs		lactate	75.2 ± 6.6^b^
		butyrate	30.1 ± 15.3^a^
		β-hydroxybuytyrate	41.4 ± 12.3^a^
		creatine	98.5 ± 20.1
		L-carnitine	74.4 ± 9.2^b^
Ketone body		acetoacetate	73.9 ± 14.6^b^
		acetone	113 ± 13

HEK293T cells were transfected with MCT7/pEGFP-C1 or empty-vector. Uptake of [^3^H]taurine by the cells was measured by Na^+^-free buffer for 30 min. The substrates/inhibitors of transporters were used at 50 mM except for homocysteic acid at 30 mM. MCT7-mediated uptake was calculated by subtracting the uptake amount of mock-transfected cells from that of MCT7-transfected cells. Each point represents the mean ± S.D. (n = 6).

a *p* < 0.01

b *p* < 0.05, compared with the corresponding control by one-way ANOVA with Tukey's multiple comparisons test.

## Discussion

Mammalian MCT7 is believed to be a ketone body transporter, whose function has not been clarified in mammalian cells. We first observed that MCT7 mediates facilitative taurine transport in mammalian cells. Taurine is a major organic osmolyte involved in not only cell volume regulation but also in antioxidant and inflammatory effects. Given that these functions exclusively depend on the cellular compartmentalization of taurine, its proper distribution to those compartments must be important in the physiology. Taurine distribution is mainly regulated by the membrane transport. Therefore, our finding of a novel taurine transport mechanism may be closely related with the physiological effect(s).

Zebrafish Slc16a6a, an ortholog of SLC16A6, transports β-hydroxybutyrate in the liver; *Slc16a6a* function-loss-mutant has been implicated in hepatic steatosis (9). In this study, we found that MCT7 transports taurine (Fig. 2, *A* and *B*). Taurine is known to decrease the accumulation of lipids (triacylglycerol and cholesterol) and attenuate the development of hepatic steatosis in the liver (20, 21, 22). However, it remains unclear whether MCT7-mediated taurine transport contributes to the pathology of steatosis. Further studies investigating the relationship between taurine transport and hepatic steatosis are warranted.

Taurine is well distributed throughout the body and highly concentrated in tissues such as neutrophils. Those local concentrations are extremely high and reached to millimolar order (23). Therefore, low affinity transport system(s) is needed for an efficient efflux of taurine from cells. In our experiments, MCT7-mediated taurine transport was nonsaturable (Fig. 2*E*). The nonsaturable transport is suitable for efflux of taurine from tissues or cells where taurine is highly accumulated. Taurine efflux from cells is known to be mediated by volume-regulated anion channel (VRAC), which consists of heterodimer of leucine-rich repeat-containing 8 family of proteins (24, 25). The efflux of taurine *via* VRAC is activated by hypoosmotic stress and nonsaturable under physiological taurine concentration. Furthermore, VRAC mediates efflux of not only taurine but other anions such as glutamic acid and aspartic acid (26). However, other molecules contributive to taurine efflux were not fully understood. Our study showed MCT7 mediates efflux of taurine from cells under normal osmotic condition (Fig. 2*C*). The MCT7-mediated taurine transport was also nonsaturable and sensitive to L-glutamate and L-aspartate (Fig. 2*E* and Table 1). These transport characteristics of MCT7 seem to be similar to those of VRAC, although it is not clear whether MCT7 is activated by hypoosmotic stress. However, taurine is generally taken up and concentrated in the cells as an osmolyte under hypertonic conditions. Subsequently, it is released from the cells under normal osmotic conditions, particularly during recovery from the hypertonic conditions. During this process, the facilitative MCT7-mediated transport of taurine might be helpful for the rapid recovery of the osmotic balance. This is because MCT7 can act as a facilitative taurine transporter under normal osmotic conditions, unlike VRAC.

Taurine is rapidly absorbed from small intestine and distributed into tissues through the blood (27, 28), although membrane permeation is extremely limited due to high hydrophilicity. In intestinal epithelial cells, TAUT/SLC6A6 and PAT1/SLC36A1 are known to contribute to taurine absorption at the lumen side (19, 29). However, to the best of our knowledge, the release mechanism of taurine to the blood side remains unknown. We showed that MCT7 enhanced taurine permeation across Caco-2 cells and was localized at the basolateral membrane of the cells (Fig. 4, *B* and *C*). MCT7 is reported to be expressed in the small intestine of rat (11). We also confirmed the mRNA expression (Fig. S3). Considering its facilitative transport property, MCT7 could function in taurine uptake as well as taurine efflux in intestinal epithelial cells. Taurine is generally obtained through diet. TAUT and PAT1 are involved in taurine absorption in the intestine, leading to the intracellular accumulation of taurine in enterocytes. Under such conditions, it seems that MCT7 performs taurine efflux rather than taurine uptake in enterocytes. Typical substrates of TAUT (β-alanine and GABA) and PAT1 (L-alanine and L-proline) did not inhibit MCT7-mediated taurine uptake (Table 1). Conversely, uptake was inhibited by monocarboxylates (β-hydroxybutyrate, butyrate, and lactate) and acidic amino acids (L-aspartate and L-glutamate). Moreover, MCT7 expression enhanced the decrease of intracellular amino acids (Fig. 1). These data suggest that MCT7 has broad substrate recognition for not only taurine but other acidic metabolites. In this study, we did not determine the nature of inhibition by acidic metabolites (homocysteic acid, aspartate, and glutamate) owing to the low affinity of MCT7 for taurine. Alternatively, we examined whether these compounds are directly transported by MCT7 *via* uptake and efflux studies and found that homocysteic acid and glutamate were transported by MCT7 (Fig. S3). However, no differences were noted in the transport of aspartate between MCT7- and mock-transfected cells, implying the noncompetitive or uncompetitive inhibition of taurine uptake by aspartate. Therefore, MCT7 may function as one of taurine releasers at the blood side of rat intestinal epithelial cells rather than a specific counterpart of TAUT or PAT1. However, in the human intestine, it remains unclear whether MCT7 can contribute to taurine absorption because there are some controversial reports regarding the expression of MCT7 in the human intestine (30, 31). In pooled tissues of the human intestine, the mRNA of MCT7 was detected by RT-qPCR, and the expression level was higher than that in the liver (30). Conversely, single-cell RNA-seq analysis showed MCT7 expression in the suprabasal cells of the esophagus, but the expression in the enterocytes of the duodenum was slight compared with that in the esophagus (31). Further research on the expression of MCT7 in the human intestine is warranted.

It is well known that MCT1, MCT3, and MCT4 interact with CD147; whereas, MCT2 mainly interacts with GP70. We showed MCT7 interactions with both CD147 and GP70 using the BiFC assay (Fig. 3*C*). MCT7, like MCT2, seems to interact preferably with GP70 than with CD147, as the potent functional regulation of MCT7 was shown (Fig. 3, *A* and *B*). Furthermore, we observed localization of EGFP-tagged MCT7 when co-expressing ancillary proteins (Fig. S5), in which the MCT7 signals seemed to be basically localized at the plasma membrane and a part of the signal showed a dotted pattern. Only the dot signals were decreased by co-expression of ancillary proteins, although it is not clear the dot signals shows mislocalized, aggregated, or internalized MCT7 protein. The interactions between MCT7 and ancillary proteins affect the MCT7 proteins that showed dotted signals, resulting in the enhancement of MCT7-mediated taurine transport. However, it has remained unclear whether these interactions occur under a physiological condition and are essentially needed for the functional expression of MCT7.

Recently, a cryo-EM structural determination study reported that MCT1 contains three critical amino acids (K38, D309, and R313) for proton-recognition in the amino acid sequence (32). The three amino acids are completely conserved among prototypical proton-driven MCTs (MCT1, MCT2, MCT3, and MCT4). However, MCT7 lacks the amino acid corresponding to D309, although the amino acids corresponding to K38 and R313 are conserved. It is reported that the substitution of D293 in MCT2 (D309 in MCT1) reduces proton transport activity (33). Thus, the pH-independent taurine transport property of MCT7 may be explained by the missing amino acid required for proton recognition in the MCT7 sequence.

In summary, our study findings demonstrate that MCT7 is a novel facilitative taurine transporter in mammalian cells. MCT7 could be a taurine releaser at the plasma membrane of cells including polarized intestinal epithelia. At least, functional expression of MCT7 is partially regulated by the interaction with ancillary protein CD147 and GP70 under *in vitro* condition. Our findings suggest a novel physiological role of MCT7 as a taurine transporter.

## Experimental procedures

### Materials

[^3^H]Taurine (specific radioactivity, 11.2 mCi/mmol), [^3^H]L-glutamate (specific radioactivity, 50.8 mCi/mmol), and [^3^H]L-aspartate (specific radioactivity, 12.3 mCi/mmol) were purchased from PerkinElmer. All other reagents were of analytical grade. Cell culture media and fetal bovine serum were obtained from Fujifilm Wako Pure Chemical and Nichirei Biosciences Inc, respectively. pMK243 (Tet-OsTIR1-PURO) and AAVS1-T2 CRIPR/pX330 were gifts from Masato Kanemaki (Addgene plasmid #72835; http://n2t.net/addgene:72835; RRID: Addgene_72,835, and #72833; http://n2t.net/addgene:72833; RRID: Addgene_72833) (34). pBiFC-VN155 (I152L) and pBiFC-VC155 were gifts from Chang-Deng Hu (Addgene plasmids #27097; http://n2t.net/addgene:27097; RRID: Addgene_27097, and #22011; http://n2t.net/addgene:22011; RRID: Addgene_22011) (16, 35).

### Construction of expression vectors

The cDNA was synthesized from Wistar rat intestines or brains using ReverTra Ace (Toyobo). The coding DNA sequence of *Slc16a6*, *CD147*, and *Gp70* was amplified from the cDNA by PCR. The PCR product was inserted into pCI-Neo vector (Promega), pEGFP-C1 (Clontech), pBiFC-VN155 (I152L), or pBiFC-VC155. To establish a stable MCT7 expressing cell line with Tet-on system, the coding DNA sequence of EGFP-fusional *Slc16a6* was amplified by PCR and inserted into pMK243 vector.

### Cell lines and culture conditions

HEK293T cells (Cat. No. American Type Culture Collection CRL-3216) and Caco-2 cells (Cat. No. American Type Culture Collection HTB-37) were used to perform transport studies. Those cells were cultured in Dulbecco's Eagle's Minimum Essential Medium, supplemented with fetal bovine serum at a final concentration of 10% and antibiotics (penicillin, 100 mU/ml; streptomycin, 100 μg/ml) with or without 1% nonessential amino acids (Thermo Fisher Scientific).

### Establishment of Caco-2 cells conditionally expressing EGFP-tagged MCT7

Caco-2 cells were transfected with the plasmid of EGFP-tagged MCT7/pMK243 and AAVS1-T2 CRIPR/pX330 by using polyethylenimine and then cultured in a culture medium containing puromycin to prepare Caco-2 cells conditionally expressing MCT7 under the control of Tet-on promoter (Caco-2-Tet-MCT7). Additionally, the puromycin-resistant Caco-2 cells were used for transport assays. The expression of EGFP-tagged MCT7 in the cells was induced by doxycycline and confirmed by fluorescence microscopy (BZ-X810; Keyence).

### Intracellular amino acid analysis

HEK293T cells were transfected with MCT7/pEGFP-C1 by using polyethylenimine and cultured for 48 h. The cells were incubated in the Na^+^-free buffer consisted of 10 mM Hepes/Tris (pH 7.4), 273 mM mannitol, 5.4 mM KCl, 4.2 mM KHCO_3_, 1.3 mM CaCl_2_, 0.44 mM KH_2_PO_4_, 0.49 mM MgCl_2_, 0.4 mM MgSO_4_, 0.34 mM K_2_HPO_4_, and 5.6 mM D-glucose for designed time. The amino acid contents of the cells were analyzed by LC-MS/MS system. The cells were scraped with ice-cold distilled deionized water, and the cell suspensions were deproteinated by adding acetonitrile. The samples were passed through a 0.45-μm PVDF filter (Captiva; Agilent Technologies), and the filtrates were used for analysis. The analysis was operated in positive ionization mode using a Waters Acquity UPLC H-Class connected with the Xevo TQD system (Milford). LC separation was performed at a 0.6 ml/min flow rate on an Intrada Amino acid analytical column (50 × 3 mm, 3 μm) (Imtakt). The separation was done by using a gradient program. The gradient program was composed of solvent A (20 mM ammonium formate buffer in water) and solvent B (acetonitrile) as follows: 10% A for 0 to 1.0 min, 10% to 100% A for 1.0 to 2.3 min, 100% A for 2.3 to 2.9 min, and 10% A for 2.9 to 3.8 min. The column temperature was set at 35 °C. Mass Lynx software, version 4.1, software was used to control the instrument and to collect data.

### Transport studies

Uptake and efflux of taurine were measured using monolayers of cultured cells in 48-well or 24-well culture plates using Na^+^-free buffer or Hank's balanced salt solution buffer (Na^+^-containing buffer). The culture plates were kept in a water bath at 37 °C. The culture medium was aspirated, and the cells were washed by each buffer. For uptake study, Na^+^-free buffer containing [^3^H]taurine was added to the cells. Following incubation for indicated periods, the medium was removed by aspiration, and the cells were washed twice with ice-cold Na^+^-free buffer. In the efflux study, [^3^H]taurine was loaded into cells using Na^+^-containing buffer for 30 min. After loading [^3^H]taurine, the cells were washed by ice-cold Na^+^-free buffer and then incubated in prewarmed Na^+^-free buffer for indicated periods. After incubation, the Na^+^-free buffer was removed. The cells were lysed in 0.1% Triton-X100/0.3 M NaOH. The lysate was used for the measurement of radioactivity and BCA protein assay.

Permeation assay was performed using Caco-2-Tet-MCT7. Caco-2-Tet-MCT7 was plated on Falcon cell culture inserts (3-μm pores, 353090, BD-Falcon). The cells were cultured in a normal culture medium for 21 days and then treated with 10 μg/ml doxycycline and 10 mM sodium butyrate for 2 days to induce MCT7 expression. The permeation of [^3^H]taurine and lucifer yellow across the cells was evaluated in an Hank's balanced salt solution buffer (pH 7.4).

### Statistics

All measurements were always made in more than triplicate, and the experiments were repeated twice with separate cultures. In all cases, data are expressed as means ± SD. Statistical analyses and graphing were performed in GraphPad Prism 9.1 software. Statistical differences between control and experimental groups were analyzed by one-way analysis or two-way analysis of variance followed by Tukey's test or Sidak's test, for multiple comparisons; *p* < 0.05 was considered significant. For these statistical tests, the normality was confirmed using the GraphPad Prism 9.1 software.

## Data availability

All data are included within the manuscript.

## Supporting information

This article contains supporting information.

## Conflicts of interest

The authors declare that they have no conflict of interest with the contents of this article.
