# Supplementary material for: Mammalian monocarboxylate transporter 7 (MCT7/Slc16a6) is a novel facilitative taurine transporter
Source: J Biol Chem. 2022 Mar 5;298(4):101800. doi: 10.1016/j.jbc.2022.101800 (PMC8980330; doi:10.1016/j.jbc.2022.101800)
Supplement: Supplemental Figures S1–S5 [file mmc1.docx]

SUPPORTING INFORMATION

**Mammalian monocarboxylate transporter 7 (MCT7/*SLC16A6*) is a novel facilitative taurine transporter**

**Authors:**

Kei Higuchi, Ryuto Tomabechi, Koki Sugiyama, Hisanao Kishimoto, and Katsuhisa Inoue

**Affiliations:**

*Department of Biopharmaceutics, School of Pharmacy,* *Tokyo University of Pharmacy and Life Sciences, 1432-1, Horinouchi, Hachioji, Tokyo 192-0392, Japan*

*
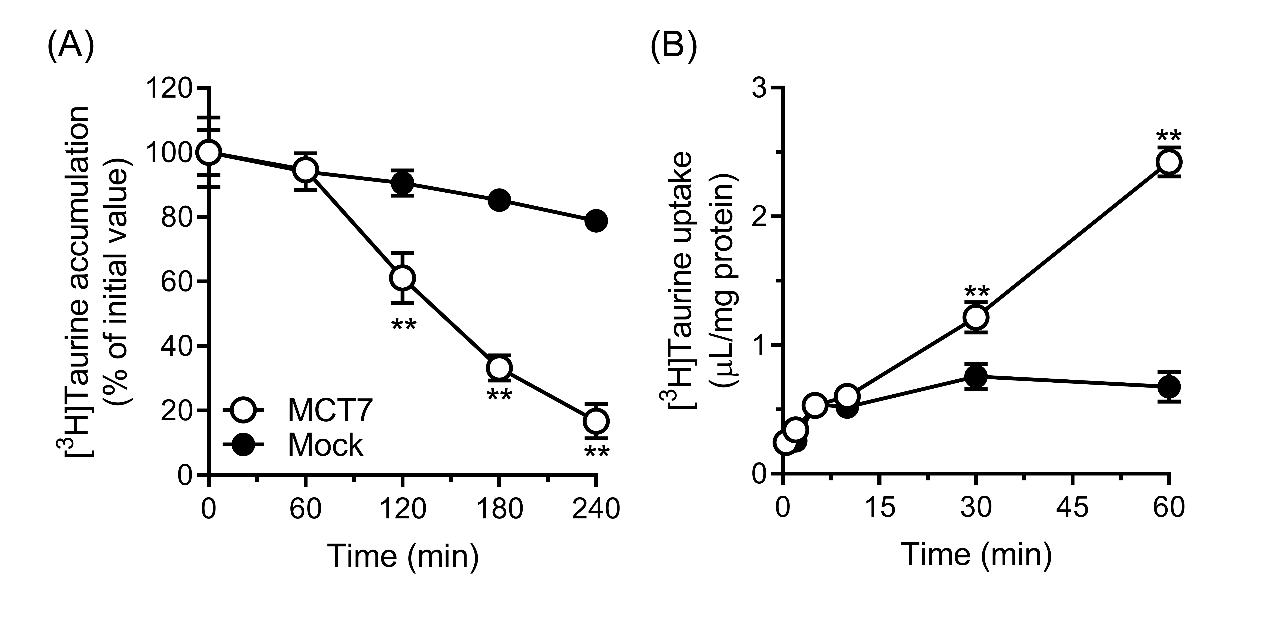
*

**Fig S1. Transport of [^3^H]taurine by HEK293T cells expressing untagged MCT7 (MCT7) or empty-vector (mock).**

(A) [^3^H]Taurine was loaded to the cells by incubation in NaCl buffer for 30 min. The cells were incubated in Na^+^-free buffer (pH 7.4) for designed time. The residual [^3^H]taurine in the cells was measured. (B) [^3^H]Taurine uptake (5 μM) was measured in Na^+^-free buffer (pH 7.4) for designed time. Each point represents the mean ± S.D. (n = 3). ** *p* < 0.01, compared to the corresponding mock-transfected cells by two-way ANOVA with Sidak's multiple comparisons test.


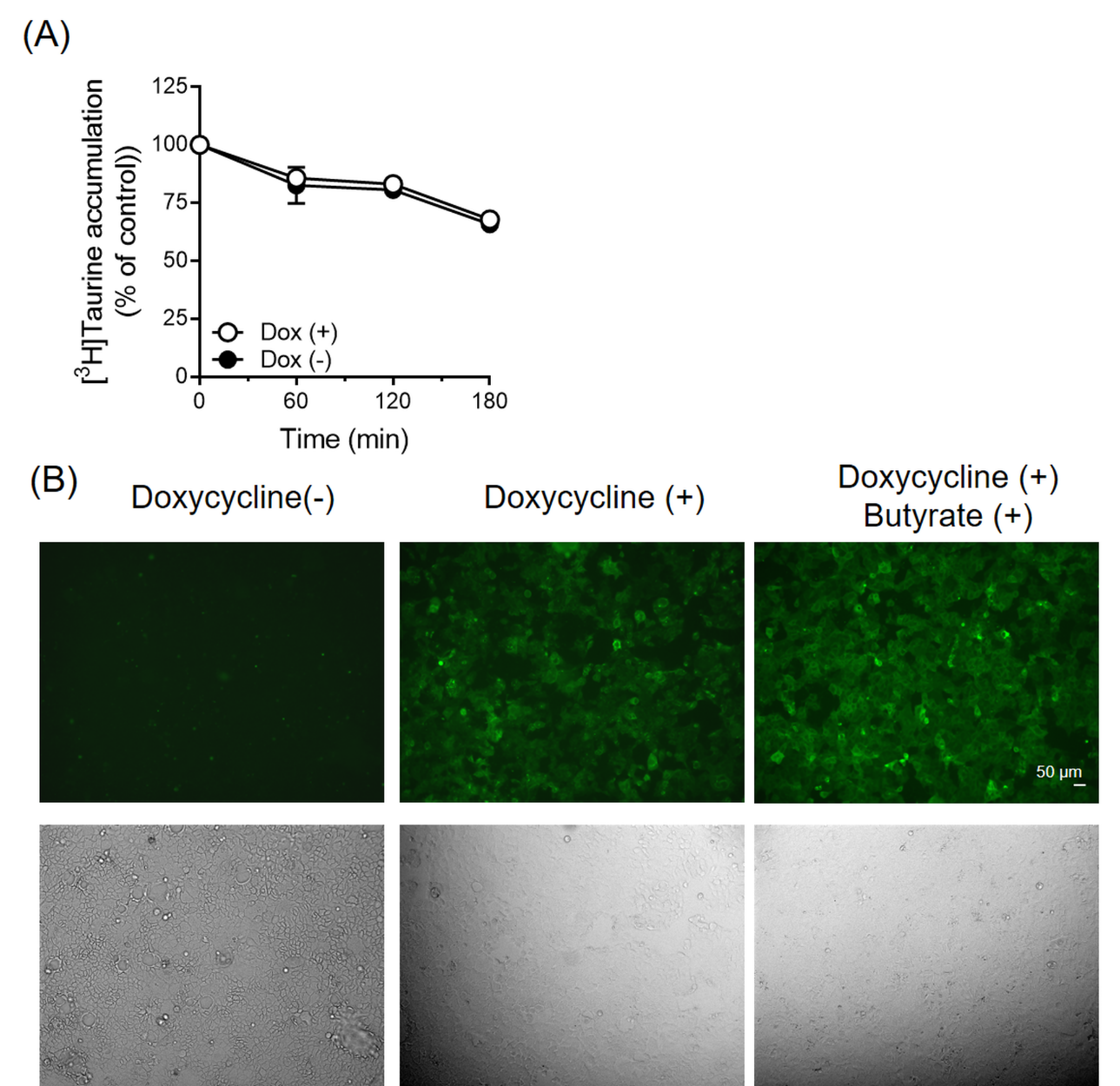


**Fig S2. Effect of butyrate on EGFP-tagged MCT7 expression in Caco-2-Tet-MCT7 cells**

(A) Efflux of [^3^H]taurine by Caco-2-Tet-MCT7 cells treated with only doxycycline (10 μg/mL) for 48 hr was evaluated. [^3^H]taurine was loaded into those cells by incubation with NaCl buffer. Those cells were incubated in Na^+^-free buffer (pH 7.4) for designed time. The residual [^3^H]taurine in the cells was measured. Each point represents the mean ± S.D. (n = 3). (B) Caco-2-Tet-MCT7 cells were cultured in normal culture medium for 2 days in a glass bottom dish. The cells were treated with sodium butyrate (10 mM) and/or doxycycline (10 μg/mL) for 48 hr. The EGFP-signal in the cells were detected by fluorescence microscopy. Upper and lower panels show GFP images and phase contrast images, respectively.


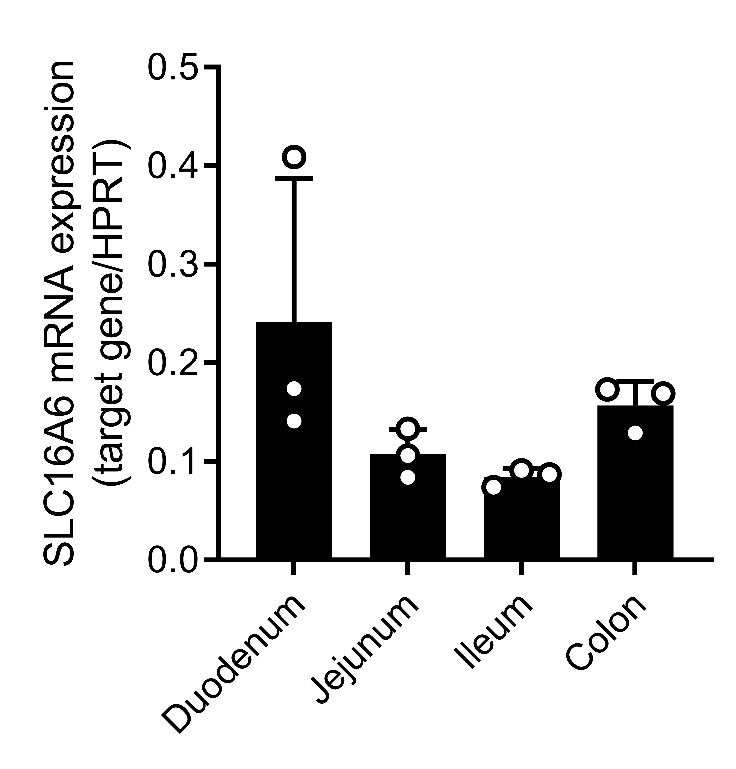


**Fig S3. mRNA expression of MCT7 in rat intestine and colon.**

Quantitative-RT-PCR was performed with Thunderbird SYBR qPCR Mix (Toyobo) using 1.3 ng of rat intestinal cDNA. Primer sequences were as follows: SLC16A6 forward, 5’-TCACTGCGGTTGCTTCTACG-3’; SLC16A6 reverse, 5’-TTCAGGGCCGTGATAGC-TGG-3’; HPRT- forward,5’-GCGTCGTGATTAGCGATGATGAAC-3’; HPRT-reverse, 5’-CCTCCCATCTCCTTCATGACATCT-3’. Amplification and detection for Quantitative-PCR were carried out on an AriaMx Real-Time PCR instrument (Agilent Technologies, Inc.), and the results were analyzed by AriaMx Software version 1.71. The relative mRNA expression was determined by the 2^-ΔΔ^ Ct method. HPRT was used as a housekeeping gene to normalize the relative expression level in samples.


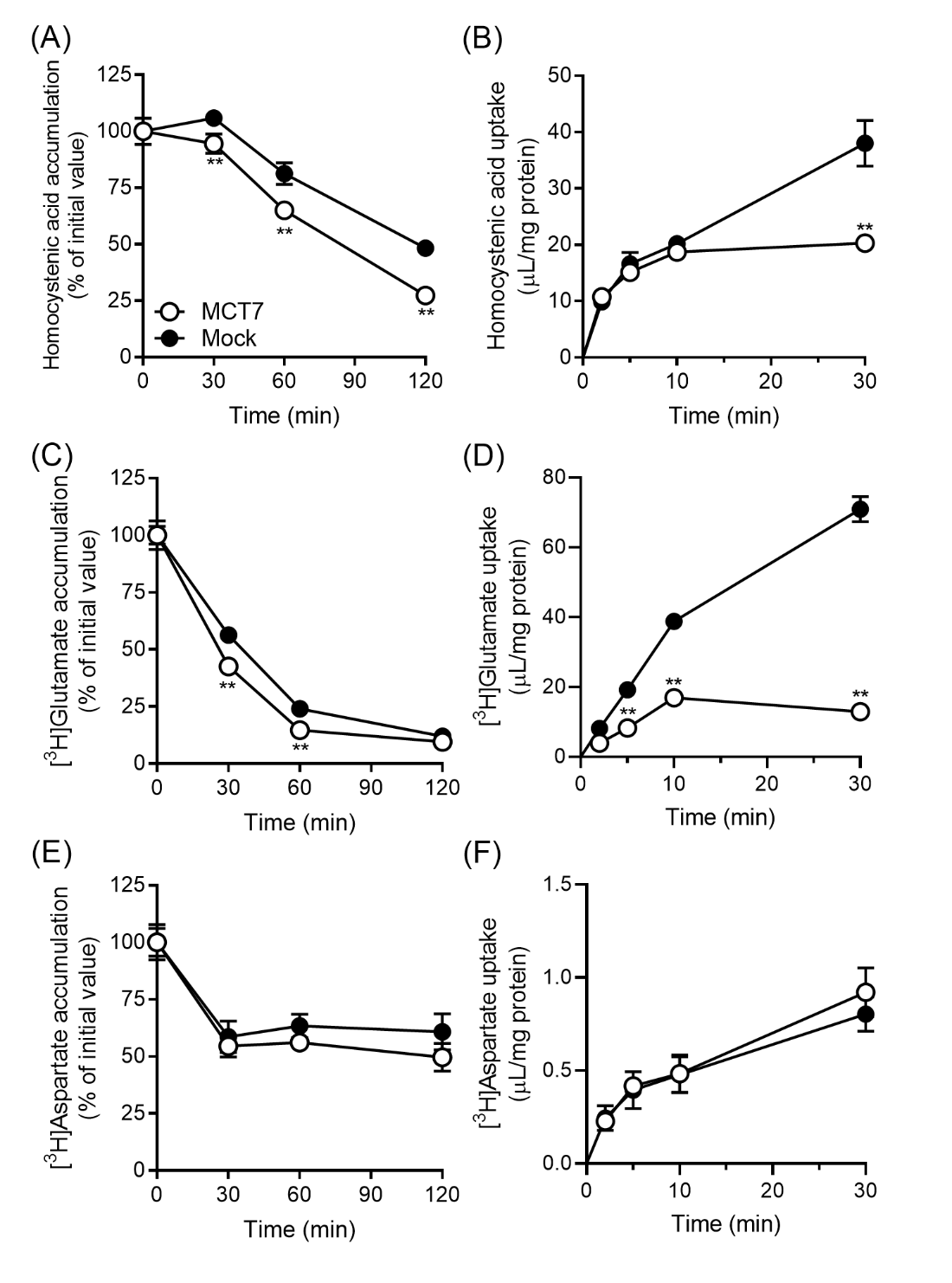


**Fig S4. Efflux and uptake of** **homocystenic acid, [^3^H]glutamate, and [^3^H]aspartate by HEK293T cells expressing EGFP-tagged MCT7 (MCT7) or empty-vector (mock).**

(A, C, E) Homocystenic acid, [^3^H]glutamate, and [^3^H]aspartate (5 μM ) were loaded to the cells by incubation in NaCl buffer for 30 min. The cells were incubated in Na^+^-free buffer (pH 7.4) for designed time. The residual compounds in the cells were measured. (B, D, F) Those uptakes (5 μM or 100 μM) were measured in Na^+^-free buffer (pH 7.4) for designed time. Each point represents the mean ± S.D. (n = 3). ** *p* < 0.01, compared with the corresponding mock-transfected cells by two-way ANOVA with Sidak's multiple comparisons test.

**
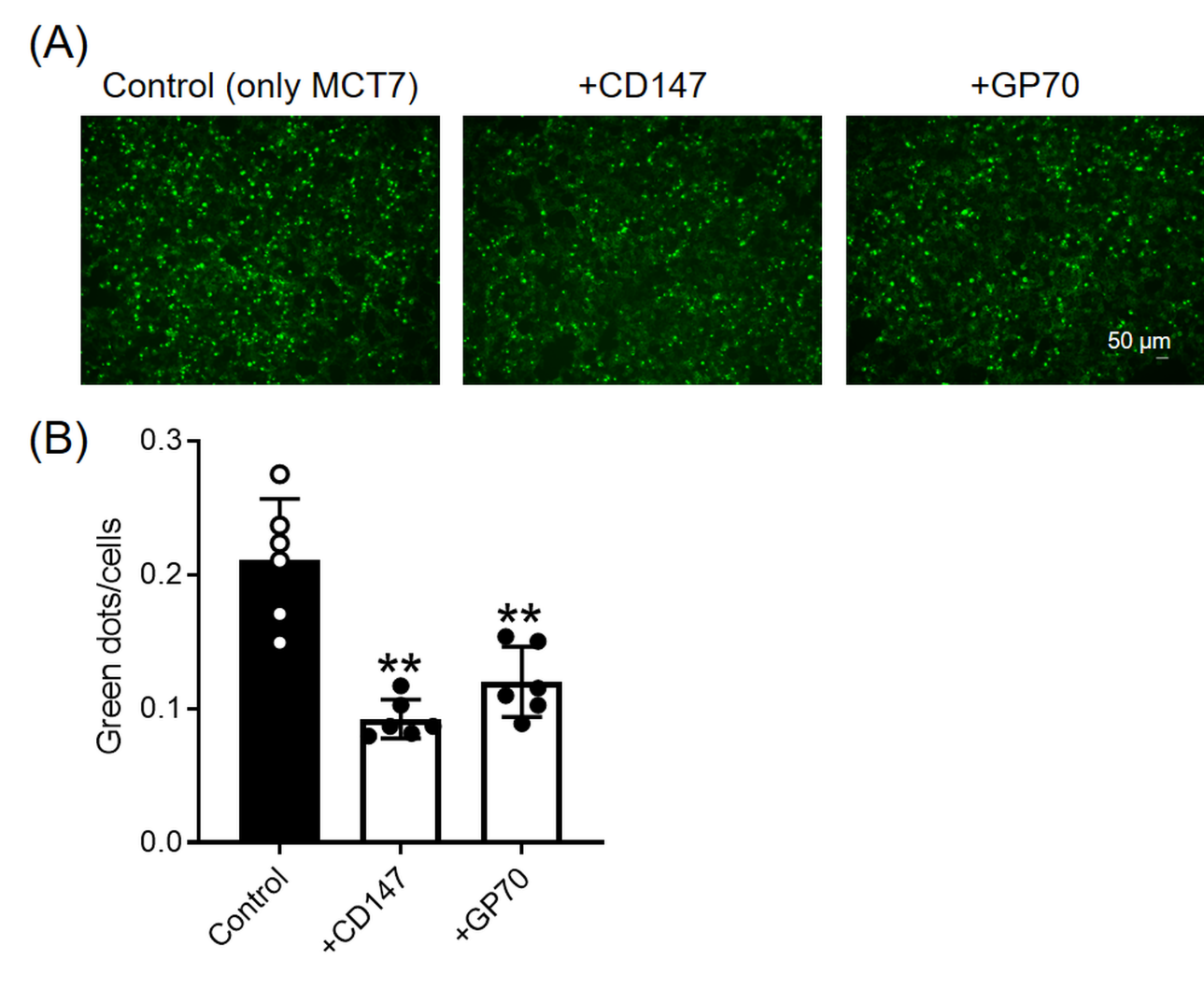
**

**Fig S5. Effect of CD147 and GP70 on MCT7 localization in HEK293T cells**

**(A)** HEK293T cells were cultured on chamber slide and transfected with EGFP-tagged MCT7 and CD147 or GP70. The cells were cultured for 48 hr and then the nuclei were stained by Hoechst 33342 for counting cell numbers. The EGFP signal in the cells were detected by fluorescence microscopy. Each representative image was shown. **(B)** The green dots were counted in each image. The counts were correlated by cell numbers. Each bar represents the mean ± S.D. (n = 6). ** *p* < 0.01, compared with the corresponding control by one-way ANOVA with Tukey’s multiple comparisons test.
